# Childhood Bullying, Paranoid Thinking and the Misappraisal of Social Threat: Trouble at School

**DOI:** 10.1007/s12310-017-9238-z

**Published:** 2017-11-22

**Authors:** Alexander H. Jack, Vincent Egan

**Affiliations:** Centre for Family and Forensic Psychology, Division of Psychiatry and Applied Psychology, School of Medicine, University of Nottingham, Nottingham, NG81BB England, UK

**Keywords:** School, Bullying, Paranoid thinking, Victimization, Cognitive bias, Threat

## Abstract

Experiences of bullying predict the development of paranoia in school-age adolescents. While many instances of psychotic phenomena are transitory, maintained victimization can lead to increasingly distressing paranoid thinking. Furthermore, paranoid thinkers perceive threat in neutral social stimuli and are vigilant for environmental risk. The present paper investigated the association between different forms of bullying and paranoid thinking, and the extent to which school-age paranoid thinkers overestimate threat in interpersonal situations. Two hundred and thirty participants, aged between eleven and fourteen, were recruited from one secondary school in the UK. Participants completed a series of questionnaires hosted on the Bristol Online Survey tool. All data were collected in a classroom setting in quiet and standardized conditions. A significant and positive relationship was found between experiences of bullying and paranoid thinking: greater severity of bullying predicted more distressing paranoid thinking. Further, paranoid thinking mediated the relationship between bullying and overestimation of threat in neutral social stimuli. Exposure to bullying is associated with distressing paranoid thinking and subsequent misappraisal of threat. As paranoid thinkers experience *real* and *overestimated* threat, the phenomena may persist.

## Introduction

Paranoia has been defined as “the unfounded fear that others intend to cause you harm” (Freeman et al., 2008, p. 258), with evidence to suggest that the phenomenon extends beyond clinical disorder and that non-clinical expressions are prevalent in the general population (Bebbington et al., 2013; Freeman et al., 2011). As such, incidence of paranoid thinking (and kindred psychotic phenomena) are noted to exist on a continuum of severity, with transient experiences of limited concern, and problematic manifestations demarcated by frequency, intrusiveness and associated distress (van Os, Linscott, Myin-Germeys, Delespaul, & Krabbendam, 2009). Indeed, consideration of distress is noted to be vital when attempting to understand the severity of experiences (Peters, Joseph, & Garety, 1999; Shevlin, Boyda, Houston, & Murphy, 2015a). Transient experiences have been observed to have greater prevalence in childhood and adolescence (Kelleher et al., 2012), though such developmental expressions rarely migrate to psychotic disorder (Dominguez, Wichers, Lieb, Wittchen, & van Os, 2011). However, troubling experiences are noted to become of increased clinical concern with greater exposure to environmental risk factors (Linscott & van Os, 2013).

There is a growing body of literature to suggest that experiences of childhood victimization precipitate the development of paranoid thinking, with greater exposure associated with expressions of heightened severity (e.g., Lataster et al., 2006; Varese et al., 2012). As such, bullying—repeated behavior, conducted with the intention of causing harm and distress, by same-age peers when a power imbalance is evident (Olweus, Limber, & Mihalic, 1999; Arseneault, Bowes, & Shakoor 2010)—has been investigated as a risk factor of interest. Resultantly, a positive association has been noted in samples of adolescents reporting on contemporaneous experiences of bullying and paranoid phenomena (Campbell & Morrison, 2007; Shakoor et al., 2015), and adults reporting on retrospective experiences of bullying and present paranoid thinking (Ashford, Ashcroft, & Maguire, 2012; Shevlin, McAnee, Bentall, & Murphy, 2015b; Valmaggia et al., 2015).

In a British prison sample, Shevlin et al. (2015b) identified that a combination of prior sexual abuse and childhood bullying predicted co-occurring paranoia and hallucinations. However, in a general population study conducted by Bentall, Wickham, Shevlin, and Varese (2012) that used the same epidemiological methodology, no significant relationship was found. Despite this, many other studies that have explored the association have indicated an effect. For example, Catone and colleagues (2015) used the 2000 and 2007 British Adult Psychiatric Morbidity Surveys and reported that experiences of bullying were predictive of persistent persecutory ideation and hallucinations. Valmaggia et al. (2015) also reported that individuals at ultra-high risk for psychosis were more likely to have been bullied; however, in their sample, exposure to bullying was associated with paranoid ideation regardless of clinical status. In adolescent samples, Campbell and Morrison (2007) reported that 14–16 year olds who perceived that they had been the victims of bullying expressed paranoid thinking to a greater degree than those who did not. Additionally, Shakoor et al. (2015) utilized a longitudinal methodology and evidenced that bullying in childhood was associated with paranoid experiences in later adolescence. Studies that have investigated a history of bullying in those with a diagnosed psychotic disorder have also found a positive and significant association (Chaudhry, 2012; Sansen, Iffland, & Neuner, 2014). In an attempt to deconstruct bullying typologies, Ashford et al. (2012) and Chaudhry (2012) both considered direct (punching, kicking, name-calling, threat-making etc.) and indirect (rumor spreading, exclusion, and other forms of relational victimization) bullying and found that both forms were associated with paranoid thinking within their samples.

Despite the described findings, certain methodological limitations have prevented definitive conclusions with regard to causality—only one study has investigated the association using longitudinal data (Shakoor et al., 2015). Further, several studies have conceptualized paranoia dichotomously (Bentall et al., 2012; Campbell & Morrison, 2007; Shevlin et al., 2015b), with the majority of those that have used continuous data omitting distress as an adjunct to measurement. As such, it has been possible to conclude a linear relationship between childhood bullying exposure and paranoid thinking, though not within a continuum of severity, with reference to experience-associated distress (i.e., a greater number of experiences does not necessarily mean greater severity of paranoia).

### Mechanisms of Association

Cognitive bias is implicated in the development of paranoid thinking (Freeman & Garety, 2014), with paranoid individuals evidenced to show an attentional bias toward threat (Bentall & Kaney, 1989; Freeman et al., 2008). As such, experiences of victimization contribute toward the development of schematic beliefs concerning apparent danger from others and the wider world (Smith et al., 2006), which, in turn, precipitate the use of an availability heuristic that explains bias toward threat (Corcoran et al., 2006) and perception of the self as vulnerable (Johns et al., 2004). This can be exacerbated when victimization, threat, and danger also occur within the family home (Radford, Corral, Bradley, & Fisher, 2013) and local neighborhood (Jack & Egan, 2016). Relatedly, Anilmis et al. (2015) reported that, in a child sample, negative schematic beliefs about the self and others mediated the relationship between bullying and psychotic-like experiences; similar findings have been reported by Campbell and Morrison (2007) and Chaudhry (2012). Further, evidence indicates that victims of childhood bullying experience depression, anxiety, and lower self-esteem (Hawker & Boulton, 2000), negative self-related cognitions (Cook, Williams, Guerra, Kim, & Sadek, 2010) and can develop a hostile attribution bias (the perception that others’ neutral actions are conducted with hostility: Dodge, 2006; Pornari & Wood, 2010). Interestingly, An et al. (2010) have noted hostile attribution biases to be associated with—and predictive of—paranoia, and Fisher et al. (2013) have described such factors to mediate the association between bullying and psychotic phenomena. A related concept—loneliness—has been reported to moderate the mediating role of peer victimization within the context of childhood threat and subordination, and psychotic experiences (Shevlin, McElroy, & Murphy, 2015c). Similarly, Kim, Lee, Yi, and Lee (2014) stated that social exclusion heightened the risk of paranoid ideation.

### Rationale

The present study investigates the association between different forms of childhood bullying victimization and paranoid thinking, with a consideration of distress as a necessary adjunct to measurement. Further, the study will explore the extent to which paranoid thinking mediates the relationship between different forms of bullying and overestimation of threat in neutral social situations. It is important to investigate these two questions as: firstly, consideration of paranoia-associated distress will add nuance to the evidence in support of a distress-based continuum model of association; and, secondly, an appreciation of how paranoid thinkers appraise social threat will add insight into the real-world impact of cognitive biases, which might precipitate a spiral toward the severe and distressing end of the paranoid continuum. Indeed, it has been argued that a paranoid thinking style might be considered an evolutionary strategy to protect oneself from further threat (e.g., a paranoid heuristic: Preti & Cella, 2010); however, there is a danger that an individual would become engulfed by *real* and *perceived* threat signals, as neutral social events are misperceived as dangerous. In such instances, transient paranoid expressions might persist and develop into psychotic disorder.

It is hypothesized that: (1) a significant and positive relationship will be found between exposure to childhood bullying and paranoid thinking (e.g., greater bullying victimization will predict more distressing paranoid thinking); (2) accumulated domestic and neighborhood victimization, loneliness, and childhood bullying will contribute to an overall predictive model of paranoid thinking; and (3) paranoid thinking will mediate the relationship between childhood bullying and perception of threat in neutral social stimuli.

## Methods

### Design and Participants

A cross-sectional study design was employed, utilizing a sample of adolescents derived from a large secondary school in the Midlands region of the UK. Senior school staff acted as gatekeepers and assessment was conducted in quiet conditions on school sites. The survey was hosted online using the Bristol Online Survey web tool (BOS; http://www.onlinesurveys.ac.uk/). Participants were required to provide demographic information and complete a series of questionnaires, which were assessed for clarity using ‘the readability test tool’ (http://read-able.com/); results indicated they were suitable for the sample. Tacit consent was required from parents, and further informed consent requested from participants.

Two hundred and thirty participants (*M*:*F* = 123:107) were recruited. Their ages ranged from 11 to 14 (*M* = 12.57 years, SD = .91 years). Specific information pertaining to ethnicity was not requested; however, the sample was predominantly White British. The University Of Nottingham School Of Medicine Ethics Committee approved the study.

### Measures

#### Childhood Adversity Checklist (CHAS; Boyda, personal correspondence)

The CHAS is a seven-item checklist of Adverse Childhood Experiences (ACEs). Participants are asked to indicate whether a given event had occurred, with the total number of ACEs reported constituting the participants’ ‘score’. The CHAS asks participants to comment on: separation of parents; long-term financial difficulties within the family; serious conflict within the family; fear of a family member; the presence of an alcoholic within the family home; and the presence of serious physical or mental health difficulties in the family home.

#### Community Assessment of Psychic Experience: Positive Symptom Scale (CAPE; Stefanis et al., 2002)

The CAPE 20-item positive scale was used in the present study. The CAPE has five factors (hallucinations, paranoia, grandiosity, delusions, and paranormal beliefs; Shevlin et al., 2015a). The CAPE positive symptom scale is based on the Peters Delusion Inventory (PDI; Peters et al., 1999) with two additional items to capture hallucinations. The CAPE has been used extensively in research investigating the psychosis continuum, and the positive scale demonstrates good reliability and validity (0.84; Mark & Toulopoulou, 2016; Stefanis et al., 2002). Participants are required to indicate the degree to which they have experienced 20 specific phenomena on a four-point Likert scale (0 = never, 1 = sometimes, 2 = often, 3 = always). If the participant responded with anything other than ‘never’ to the initial question, they were then asked to rate the associated distress on a further four-point Likert scale (0 = not distressed, 1 = a bit distressed, 2 = quite distressed, 3 = very distressed).

#### Gatehouse Bullying Questionnaire (Bond, Wolfe, Tollit, Butler, & Patton, 2007)

The Gatehouse Bullying Scale is a 12-item questionnaire designed to measure direct and indirect bullying, designed for a target population of 11–15 year olds. It contains four questions on distinct forms of bullying, which are further broken down into subscales to measure severity and frequency. Follow-up questions are only asked if the initial overarching item is endorsed, with each measured on a three-point scale (e.g., how upsetting was it when you were teased: (0) not at all; (1) a bit; (2) I was quite upset). The four question domains related to: teasing/name-calling, rumor spreading, exclusion from peer groups, and physical violence/threat. The Gatehouse Bullying Questionnaire has demonstrated good reliability and validity (Bond et al., 2007).

#### Neutral Social Vignettes

Eight neutral social vignettes were utilized in order to assess threat perception to neutral stimuli. Each vignette was designed to be realistic, mundane and internally consistent, in line with the recommendations of Wason, Polonksy, and Hyman (2002). The vignettes were based on a format developed by Jack and Egan (2016), though were constructed of novel items specific to the aims of the present study. Each individual measure consisted of a neutral situational statement such as: ‘*someone in your school year has tagged you in a Facebook post. You are not close friends*’; a neutral statement: ‘*based on this statement*, *use the scale to rate how much you agree with the following statement*’; and a possible conclusion: ‘*the person has written something to upset/humiliate you*’. Jack and Egan (2016) reported an overall Cronbach’s alpha of 0.78 in their study.

#### Other Factors of Relevance

One item on loneliness was included, as this has been identified as a contributing factor to developing psychoses (Shevlin et al., 2015c). The question asked participants to respond, “not true”, “somewhat true”, or “certainly true” to the statement: “I feel lonely or isolated from other people”. Further, participants were asked to describe the subjective dangerousness of their neighborhood on a four-point Likert scale (1 = very safe, 2 = quite safe, 3 = quite dangerous, and 4 = very dangerous). A previous study (Jack & Egan, 2016) had demonstrated that a small yet significant proportion of variance in paranoia was explained by such perceived neighborhood danger.

#### Procedure

Questionnaires were hosted on a research website using the BOS survey building tool. The BOS system automatically logs participant responses, which can be extracted for data analysis.

In order to recruit participants, a standardized email was sent to secondary schools in the Midlands region of the UK with the aim of agreeing collaboration. If no response was forthcoming, a follow-up telephone call was made. In total, 10 secondary schools were contacted. Only one school agreed to facilitate the project. The survey was administered during designated lessons within the school day, facilitated by research volunteers and supervised by teaching staff. Participating classes were chosen by school gatekeepers, and, as classes were not arranged by academic attainment, a range of abilities were represented in the cohort.

#### Treatment of Data

Results were extracted from the BOS system and imported into the Statistical Package for Social Sciences (SPSS) 22.0 for analysis. The data were cleaned, with four participants removed from the dataset due to invalid response patterns, which were identified by three validity questions embedded within the survey. Following this, composite scores were constructed for the CAPE and GBS using SPSS syntax commands. The CAPE composite score was constructed by combining presence and distress scores for each item before computing a total score, which has been shown to be a viable methodology (Boyda, 2014). A paranoia scale was then constructed using items from the CAPE shown to load to the factor in previous research (Boyda, 2014; Shevlin et al., 2015a). Composite scores for the GBS were constructed by adding presence, distress and frequency scores before a scale score was constructed for overt (teasing and physical threat), covert (rumor spreading and social exclusion), and total bullying.

Neutral social vignettes were coded from − 2 (strongly disagree) through 0 (neutral) to 2 (strongly agree); a total score was then calculated across the eight items. Scale scores of below 0 could be interpreted as indicative of an optimism bias, whereas scores over 0 were indicative of over-perception of threat.

## Results

### Descriptive Statistics

All measures were found to be reliable, with Cronbach’s alpha acceptable for all tasks (Table 1). Skewness and Kurtosis fell within desired parameters and were satisfactory for all measures.

**Table 1:** Descriptive statistics for all scales

	Cronbach’s alpha	Mean	SD
CAPE Paranoia Scale	.81	5.83	5.41
Gatehouse Bullying Scale	.80	6.88	6.13
Social Perception Vignettes	.90	− 2.85	7.51

Cronbach’s alpha, mean and standard deviations for measures utilized in the present study

### Hypothesis One

To investigate whether exposure to childhood bullying predicted paranoid thinking, a simple linear regression was conducted. The regression equation was significant, *F*(1, 224) = 120.934, *p* = .001, and the findings indicated that exposure to childhood bullying did predict paranoid thinking, *β* = 0.522 [95% CI .429, .616], *t*(224) = 10.997, *p* < .001. Exposure to childhood bullying predicted 34.8% of the variation in outcome scores (adjusted *R* ^2^ = .348).

### Hypothesis Two

In order to investigate the extent to which covert and overt bullying, gender, and victimization in the home and neighborhood influence paranoid thinking, a hierarchical linear regression was conducted. Gender was entered at stage one, domestic ACEs, self-reported dangerousness of local neighborhood, and loneliness were entered in stage two, and covert and overt bullying were entered in stage three (Table 2).

**Table 2:** Hierarchical linear regression to determine predictors of paranoid thinking

	Model one				Model two				Model three			
	*B*	SE *B*	*β*	*p*	*B*	SE *B*	*β*	*p*	*B*	SE *B*	*β*	*p*
Gender	1.937	.713	.240	<.001	1.974	.580	.183	.001	2.018	.562	.187	<.001
Domestic ACEs					.835	.213	.217	<.001	.397	.216	.103	.068
Perceived dangerous neighborhood					1.113	.487	.125	.023	.996	.459	.112	.031
Loneliness					2.257	.449	.450	<.001	2.257	.493	.280	<.001
Overt bullying									.336	.118	.201	.005
Covert bullying									.313	.119	.192	.009
*R* ^2^	.057				.399				.473			
*R* ^2^ change					.341				.074			

Hierarchical linear regression to determine predictors of paranoid thinking

The findings indicated that at stage one, gender (being female) contributed significantly to the regression model, *F*(1, 216) = 13.15, *p* < .001, and accounted for 5.7% (*R* ^2^ = .057) of the variation in paranoid thinking. At the second stage, domestic ACEs, self-reported dangerousness of local neighborhood, and loneliness were introduced to the model and accounted for a further 34.1% of variation in paranoid thinking. This change was also significant, *F*(4, 213) = 35.28, *p* < .001. At the final stage, the addition of overt and covert bullying explained an additional 7.4% of variation in paranoid thinking, with the regression equation again significant, *F*(6, 211) = 31.51, *p* < .001. In the final model, 47.3% of variation was accounted for by the included variables. However, the domestic ACE score was not a significant contributor to the model.

### Hypothesis Three

**Fig. 1:**
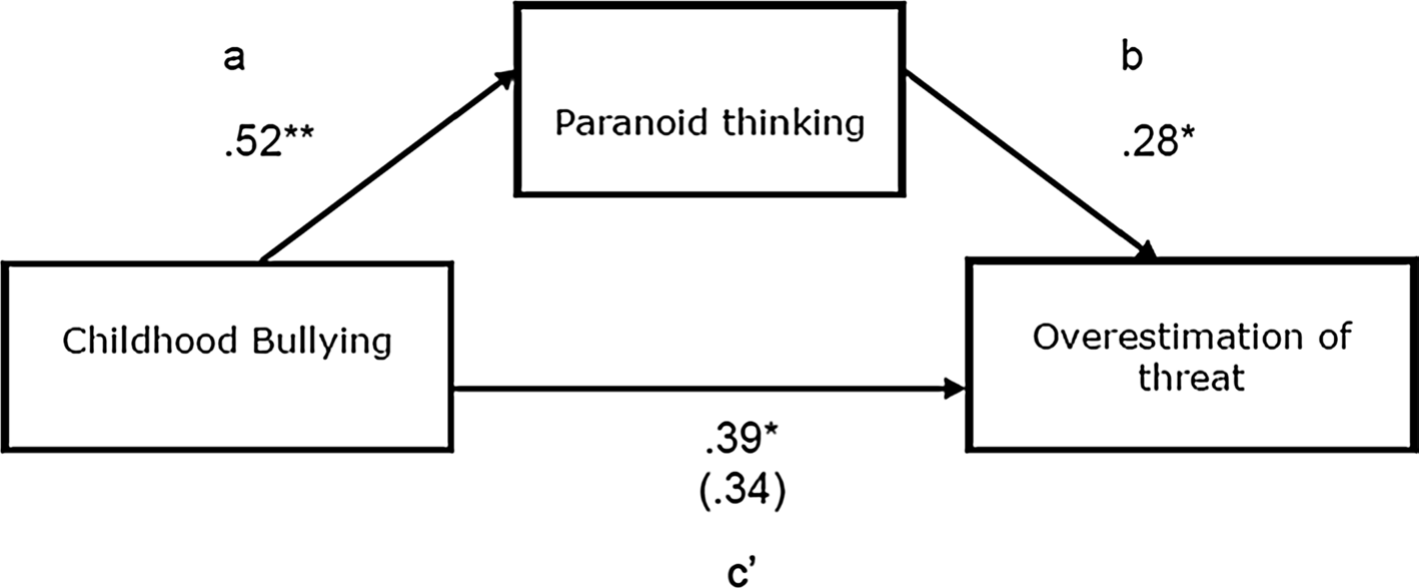
Diagram depicting mediating role of paranoid thinking on the relationship between childhood bullying and overestimation of threat

To establish whether paranoid thinking mediated the relationship between experiences of childhood bullying and overestimation of threat, a mediation analysis was conducted using the PROCESS Macro for SPSS (Hayes, 2015). Results indicated that there was a significant indirect effect of childhood bullying on overestimation of threat in neutral social stimuli through paranoid thinking, *ab* = .14 BCa 95% CI = .03 to .27. Paranoid thinking could account for approximately half of the total effect, *P*_*M*_ = .43 (Fig. 1).

## Discussion

The results of this study indicate that exposure to childhood bullying is positively associated with paranoid thinking, which is generally supportive of the existing literature (Campbell & Morrison, 2007; Catone et al., 2015). Further, as the severity of bullying increased, so did the distress associated with paranoid thinking (Shakoor et al., 2015; Valmaggia et al., 2015).

Both covert and overt bullying were significant predictors of paranoid thinking, with overt bullying demonstrating a slightly stronger effect. When social and environmental factors were considered, being female, domestic ACEs, living in a perceived dangerous neighborhood, and loneliness also contributed substantially to the best predictive model. Lastly, paranoid thinkers perceived threat in neutral social stimuli, again supportive of previous research (Freeman et al., 2008; Jack & Egan, 2016). Paranoid thinking was shown to strongly mediate between experiences of childhood bullying and overestimation of threat.

Cognitive bias is implicated in the development of paranoid thinking (Freeman & Garety, 2014). Smith et al. (2006) have argued that experiences of victimization contribute toward the development of schematic beliefs concerning danger, and Corcoran et al. (2006) have identified that such internalized beliefs manifest as an availability heuristic when paranoid thinkers appraise situations for threat; this has also been demonstrated in a sample of bullied adolescents (Anilmis et al., 2015). The results of the present study appear to uphold these findings. Further, Johns et al. (2004) have reported that experiences of victimization precipitate a sense of the self as vulnerable.

Experiences of bullying often have a deleterious effect on the formation of adolescent self-concept and their perception of the world around them (Hawker & Boulton, 2000; Cook et al., 2010). It is plausible that bullied adolescents experience a sense of vulnerability and anxiety concerning their peer relationships, which precipitates and maintains factors foundational to the formation of persecutory beliefs. In the present study, loneliness was found to be a significant contributor to paranoid thinking. Shevlin et al. (2015c) reported that loneliness moderated the mediating effect of peer victimization on the relationship between ACEs and psychosis. When concluding, Shevlin and colleagues make reference to the ‘loneliness loop’ (Hawkley & Cacioppo, 2010), which implies that feelings of loneliness, possibly exacerbated by experiences of social exclusion and rumor spreading captured within the present study, may trigger hypervigilance toward the social environment, which precedes the development of problematic cognitive biases. This hypothesis would appear congruent with the finding that covert bullying, constructed through social exclusion and rumor spreading, was a predictor of paranoid thinking, as experiences of social alienation are a fertile breeding ground for a sense of powerlessness, the formation of cognitive bias, and a vulnerable sense of self. Overt bullying represents a visceral challenge to social status and personal security.

A continuum interpretation of the phenomenon might be explained by the aforementioned cognitive factors. It is possible that as the severity of bullying increases, the bullied adolescent becomes at greater risk of internalizing negative schema concerning interpersonal threat, and forming cognitive biases—such as a hostile attribution bias—that precede persistent appraisals of neutral situations as threatening. Without adequate support, or challenges to misappraised perceptions of threat, the adolescent might be cast into a vicious cycle whereby instances of *perceived* victimization sit alongside *real* victimization and further reinforce core schematic beliefs and paranoid thoughts. In such instances, a paranoid thinking style might be considered an evolutionary strategy to protect the individual from further threat (e.g., a paranoid heuristic: Preti & Cella, 2010); however, this adaptive strategy might quickly become maladaptive once removed from *real* threat, with the individual becoming at risk of spiraling toward defensive impairment.

Living in a dangerous neighborhood has been shown to increase the risk of paranoid thinking styles (Jack & Egan, 2016). Within the present study a similar effect was found, which might be indicative of accumulative victimization (Radford et al., 2013) and exposure to threat both in and outside of school. Indeed, domestic ACEs also predicted paranoid thinking. This type of exposure might reinforce schematic beliefs concerning danger and exacerbate hypervigilance and cognitive bias. Further, it is also plausible that danger in the community relates to the continuation of childhood bullying outside of the school gates or through electronic media.

Interestingly, girls were found to experience distressing paranoid thinking to a greater extent than boys, which replicates the findings of Wigman et al. (2011). This finding is potentially indicative of the greater social pressures that teenage girls experience. Research finds female bullying tends to be covert and indirect (Crick & Grotpeter, 1995), and this was a significant predictor of paranoid thinking in the present study. It is plausible that teenage girls are particularly vulnerable to negative social schema and subsequent appraisals, which enhance the risk of paranoid thinking styles in relation to social status.

## Limitations

There are a number of limitations to the present study. Firstly, the study was cross-sectional by design. As such, it is not possible to comment on issues of causation. Secondly, the study made use of self-report questionnaires. Such methods have been found to be acceptable in studies investigating psychotic phenomena (Allardyce, Suppes & van Os, 2007) and bullying (Cornell & Bandyopadhay, 2009) in adolescents; however, there is the possibility that misunderstandings or inaccuracy of data can occur. To optimize data integrity, a series of validity items were included to prevent random or exaggerated response styles. While self-report questionnaires are a valid methodological approach, future studies might make use of corroborative teacher or peer reports of behavior, particularly in relation to bullying.

Only one school agreed to participate in the present study. As such, caution should be applied when attempting to generalize the results. A related limitation concerns the measurement of ACEs. Varese et al. (2012) have identified that specific early experiences of neglect and abuse are associated with paranoid thinking; however, in the present study, the school gatekeepers opposed measurement of such experiences. It is therefore possible that unreported ACEs may have acted to confound results in the present study, though it is important that this possible effect is not overstated. Further, this is a problem that is likely to occur in any research that does not control for all known effecting variables.

## Clinical Implications and Future Directions

The findings of the present study are clinically important. Professionals working with young people must consider bullying as a potential risk factor for paranoid thinking styles, and ensure that early-intervention strategies (and effective anti-bullying policies in school settings) are available to identify the phenomenon, and prevent the migration of transient experiences to distressing and impairing symptoms.

It would be advantageous for future research to be directed at further understanding the relationship between specific types of bullying and paranoid thinking. Greater awareness of these distinct trajectories could lead to targeted interventions, and advance relevant cognitive models. Further, the behavioral consequences of the toxic mix of bullying, paranoid thinking, and misappraisal of threat is an important avenue for exploration. It will be important that future studies are prospective by design, so that causation can be determined.
